# Prospective Longitudinal Study of Gait and Balance in a Cohort of Elderly Essential Tremor Patients

**DOI:** 10.3389/fneur.2020.581703

**Published:** 2020-11-13

**Authors:** Hollie Dowd, Maria Anna Zdrodowska, Keith H. Radler, Tess E. K. Cersonsky, Ashwini K. Rao, Edward D. Huey, Stephanie Cosentino, Elan D. Louis

**Affiliations:** ^1^Division of Movement Disorders, Department of Neurology, Yale School of Medicine, Yale University, New Haven, CT, United States; ^2^Department of Rehabilitation and Regenerative Medicine (Program in Physical Therapy), Gertrude H. Sergievsky Center, College of Physicians and Surgeons, Columbia University, New York, NY, United States; ^3^Taub Institute for Research on Alzheimer's Disease and the Aging Brain, College of Physicians and Surgeons, Columbia University, New York, NY, United States; ^4^Department of Neurology, College of Physicians and Surgeons, Columbia University, New York, NY, United States; ^5^Department of Neurology, University of Texas Southwestern, Dallas, TX, United States

**Keywords:** Essential Tremor, longitudinal, gait, balance, clinical

## Abstract

**Background:** Essential tremor (ET) encompasses a variety of features, including tremor, cognitive dysfunction, and gait and balance impairments. Gait and balance impairments in ET are often mild, but they can be severe and are, in some cases, associated with functional sequelae in terms of increased fall risk and reduced balance confidence. Previous research on gait and balance in ET has been limited to cross-sectional comparisons. There have been no longitudinal studies or prospective studies. As such, our understanding of natural history and possible predictors of declines in ET-related gait and balance impairments is incomplete.

**Objectives:** We (1) present natural history data on the change in gait and balance measures over time, (2) provide estimates of annual rate of change in each gait and balance metric, and (3) examine the relationship between baseline clinical predictors and changes in gait and balance over time.

**Methods:** 149 ET participants (mean age 78.7 years), enrolled in a prospective, longitudinal, clinical-pathological study, underwent an extensive evaluation of cognition, tremor, and gait and balance at three distinct intervals performed every 18 months. Gait and balance measures included a combination of performance-based tests (e.g., tandem gait, tandem stance) and self-reported assessments (e.g., number of falls, use of a walking aid).

**Results:** Between the baseline and final assessments, numerous balance and gait measures showed evidence of decline and annual rates of change were quantified for each. We examined the predictive utility of clinical variables at baseline for five gait and balance outcomes, with global cognition and executive function standing out as the most consistent predictors.

**Conclusions:** We present a much-needed look into the course of disease for elderly patients with ET, focusing on changes observed in gait and balance and the predictors of these changes. These results also add another dimension to the relevance of cognitive impairment observed in ET; such impairment can now be viewed as predictive of poorer gait and balance over time in ET. These findings are a useful tool for clinicians, patients, and their families to better understand and plan for changing disease-features over time.

## Introduction

Essential tremor (ET), the most prevalent tremor disorder, is a progressive neurological disease that affects up to 6.3% of the population aged 65 years and older (1). Historically, ET has been considered a benign, mono-symptomatic disorder with the single characterizing feature of tremor (2). However, it is now understood that ET patients can have other motor and non-motor symptoms and signs (3–5). Impairments in gait and balance have been documented across numerous studies (6–8), as have problems with cognition (9, 10), sleep (11, 12), and mood (4, 13). Gait and balance impairments in ET are often mild, but in some patients they can be severe; furthermore, in some cases they are associated with functional sequelae in terms of increased risk of falls and reduced balance confidence (8, 14–16).

Impaired gait and balance have been studied to a limited extent in patients with ET, and these impairments associated with cranial tremor and poorer cognition in ET (17–19), yet all previous research on gait and balance in ET has been limited to cross-sectional designs. There have been no longitudinal studies and no prospective studies assessing (1) natural history, (2) rate of change in gait and balance over time, or (3) the predictive utility of baseline clinical features for future gait and balance outcomes. The natural history and predictors of ET-related gait and balance impairments need to be systematically examined. We sought to bridge this knowledge gap.

Participants with ET were enrolled in a prospective, longitudinal study. In these analyses, which used data from three discrete time intervals, we (1) present natural history of change in gait and balance measures over time, (2) provide estimates of annual rate of change in each gait and balance metric, and (3) examine the predictive utility of baseline clinical predictors for subsequent changes in gait and balance over time. Broadly, the baseline predictors of interest were tremor severity, cognition, daytime sleepiness, capacity to perform activities of daily living, mood, and alcohol use. More specifically, based on existing data in ET, in the elderly, and in individuals with other neurological diseases of late life, *a priori* we hypothesized that baseline features such as higher total tremor score (20) and cranial tremor score (17); lower global cognition, (18) higher Clinical Dementia Rating (CDR) (18, 21); worse aggregate scores in the cognitive domains of executive function (22, 23), attention (22, 24) and visuospatial skills (25); greater daytime sleepiness (26); lower ability for daily activities (27); and finally, higher levels of depression, (28) and anxiety (29) and alcohol use (30) could function as predictors of long-term gait and balance impairment or greater decline in gait and balance in ET participants.

## Methods

### Recruited Participants

Two hundred and thirty participants from across the United States were enrolled in the Clinical-Pathological Study of Cognition in Essential Tremor (NINDS R01NS086736), a prospective, longitudinal study of cognitive function in ET. The study, which began in July 2014, recruited participants through the International Essential Tremor Foundation (IETF) as well as other study websites. As described previously (31, 32), participants who met the following eligibility criteria were recruited: (1) diagnosis of ET, (2) minimum age of 55 years, (3) no brain surgery for the treatment of ET, (4) willingness to participate in testing and enroll as a brain donor. All portions of the study were approved by the Yale University and Columbia University Internal Review Boards and all participants signed informed consent upon enrollment.

### General Evaluation

The evaluation of all participants was performed by thoroughly trained research associates. Initial assessments were completed at Time 1 (T1), then follow-up assessments at Time 2 (T2), and final assessments at Time 3 (T3), at intervals of ~18 months. At each assessment, clinical and demographic information, including a detailed tremor history, was collected using structured questionnaires. Details regarding the comprehensive videotaped neurological examination and neuropsychological evaluation, performed at each assessment, are also presented further below. In addition, several other assessments were performed. Participants were asked to report their general level of daytime sleepiness using the Epworth Sleepiness Scale (ESS) (33). The ESS asks participants to rate on a scale from 0 to 3 how likely they would be to doze off or fall asleep in eight situations (0 = would never doze, 3 = high chance of dozing), with total ESS scores ranging from 0 to 24 (34). The participant's self-reported competence in activities of daily living such as using a telephone, doing laundry, and handling finances, were measured using the Instrumental Activities of Daily Living Scale (IADL) (35). Participants' responses were scored on a 0−1 scale, with zero reflecting limited function for that activity. Total scores for the IADL range from 0 to 8. Depression and anxiety were measured with the Geriatric Depression Scale Thirty-Item (GDS-30) (36) and the General Anxiety Disorder Seven-Item (GAD-7) (37) test, respectively. The GDS-30 uses 30 questions in which each is rated 0 (no; symptom absent) or 1 (yes; symptom present). Total scores for the GDS-30 range from 0 to 30, with a cutoff score of 10 indicating the presence of significant depression. The GAD-7 consists of seven questions with each answer scored on a scale from 0 to 3, with increasing scores indicating greater symptom severity. Total GAD-7 scores range from 0 to 21, and a score of 8 or more indicates the likely presence of an anxiety disorder. Alcohol use was self-reported based on how many drinks the participant reported to consume in an average week during their adult life. Participants were identified as drinkers if they consumed greater or equal to one drink per week on average during their adult life. Current medications were also documented, including those with the potential to impair balance and gait (e.g., benzodiazepines, hypnotics, opioids, central analgesics, and anticonvulsants). For each participant, the number of such types of medications was counted and reported. Because of constraints in the personal environment, health concerns, and time restrictions of the home visit, certain tests were omitted for certain participants.

As in our previous studies (14), each participant underwent a comprehensive videotaped neurological examination at each assessment interval, which included a detailed evaluation of various types of tremor. A senior movement disorders neurologist (E.D.L.) reviewed all videotaped examinations and confirmed each participant's ET diagnosis using reliable and valid diagnostic criteria for ET (moderate or greater amplitude kinetic tremor on ≥ 3 tests, or head tremor in the absence of Parkinson's disease [PD] or dystonia or other causes) (38). Severity of postural and kinetic tremors was rated (0−3), resulting in a total tremor score (0−36), which is a measure of the severity of action tremor (39). A cranial tremor score (0−3) was calculated for each participant based on the number of locations (neck, jaw, voice) in which cranial tremor was present on examination (17). Intention tremor was identified during 10 repetitions of the finger-nose-finger maneuver. As described previously, intention tremor was considered present if the participant either had a score of 0.5 (probable intention tremor) in both arms or a score of 1 (definite intention tremor) in at least one arm (40).

### Neuropsychological Evaluation

The neuropsychological evaluation at each assessment comprised a comprehensive in-person cognitive test battery in each participant's home, a telephone interview with an informant (a close family member or friend of the participant), and subjective written summaries from the trained research associates summarizing qualitative observations regarding cognitive and functional abilities for each participant. The cognitive test battery excluded tests whose scores relied on the accuracy of motor responses so as not to disadvantage participants with more severe tremor (31, 32). The battery included two global cognitive screening measures [Mini-Mental State Examination (MMSE) (41) and Montreal Cognitive Assessment (MoCA) (42)] as well as multiple tests per cognitive domain as follows: *attention* [Wechsler Adult Intelligence Scale (WAIS) Digit Span subtest (43) and Symbol-Digit Modalities test (44)], *executive function* [Delis-Kaplan Executive Function System (D-KEFS) (45) Verbal Fluency, Color-Word Interference, Sorting, and Twenty Questions], *language* [Boston Naming test (46) and the Multilingual Aphasia Examination Token subtest (47)], *memory* [California Verbal Learning Test – II (48), Wechsler Memory Scale (WMS) Verbal Paired Associates (49), and WMS Logical Memory subtest A (50)], and *visuospatial function* [Judgement of Line Orientation (51), Benton Facial Recognition (52), and WAIS Visual Puzzles subtest (50)]. Aggregate scores for each cognitive domain were calculated as an average of the z-scores for each test. Z-scores of ≤ −1.5 were considered impaired.

During case conferences with collaborating expert neuropsychologists and neuropsychiatrists (E.D.H., S.C.), the neuropsychological evaluations were used to classify participants into cognitive groups (31, 32, 53). Classifications were assigned based on results from the clinical questionnaire, neuropsychological test battery, informant interview, and research associate impressions. Using these measures, each participant was assigned a rating from the CDR scale, ranging from zero to three (0 = no dementia, 0.5 = questionable dementia, 1 = mild dementia, 2 = moderate dementia, and 3 = severe dementia) (54, 55). Participants were also assigned one of three primary cognitive diagnoses: normal cognition, mild cognitive impairment (MCI), or dementia. The criteria for assigning these three cognitive diagnoses has been described previously (31). *Normal cognition* included: No impairment (CDR 0, no impairment on any test); Impairment of unlikely clinical significance (CDR 0, impairment on 1 test); Impairment of possible clinical significance (CDR 0 or 0.5, impairment on ≥ 1 test but not meeting operational criteria for MCI); Questionable or isolated functional impairment (CDR 0.5, no impairment on any test). *MCI* was defined as a CDR of 0.5 and impairment on 2 MCI-designated tests (31). *Dementia* was defined as a CDR ≥ 1 and impairment in multiple cognitive domains.

### Gait and Balance Evaluation

As a part of the videotaped neurological examination, participants were asked to complete two gait and balance tasks: tandem gait and tandem stance. In tandem gait, patients were asked to walk 10 steps on a straight path with the heel of the leading foot touching the toe of the following foot. The number of missteps, defined as the number of steps off of the straight line, was the reported outcome (6, 56). During tandem stance, the participant was instructed to stand with the heel of one foot touching the toe of their other foot and hold the position for 10 s. The reported outcome was the number of seconds the participant could hold the position. Additionally, we administered the Transformed Six-Item Activities of Balance Confidence Scale (ABC-6), an abbreviated version of the full Activities of Balance Confidence Scale (57). For each item, participants were asked to rate their confidence in performing six activities (e.g., walk on icy sidewalks, step onto or off of an escalator without using the handrails) without losing their balance or becoming unsteady on a scale from 0 (not at all confident) to 100 (completely confident). The total score ranged from 0 to 600. Participants reported how many falls and near falls, in which they felt like they were going to fall but did not actually fall, they had in the past year and past month. We also inquired whether each participant used a walking aid, including a cane, walker, or wheelchair, inside or outside of the home.

### Final Sample

Forty-seven out of 230 enrolled participants did not receive all three assessments. Of these 47 participants, 34 had died and 13 had withdrawn from the study before all assessments could be completed. This left 183 of 230 participants. Next, only participants with a confirmed ET diagnosis at every assessment interval, based on the videotaped neurological examinations assessed by E.D.L., were included. We therefore excluded 34 participants who received an alternative diagnosis or whose ET diagnosis was not confirmed at any of the three intervals (6 ET-PD, 4 dystonia, 5 who did not meet the criteria for an ET diagnosis, 19 with no videotaped neurological examination). As a result of these exclusions (total = 81), 149 of 230 enrolled participants were eligible for analyses.

### Statistical Analyses

Statistical analyses were performed using SPSS (Version 26; Chicago, IL, USA). We report demographic and clinical characteristics of participants, both at baseline and at the final assessment (Table 1). Second, we report gait and balance variables at both time intervals, the change in these variables over time, the annual rate of change in these variables, and a statistical comparison between baseline and final values of these variables (Table 2). Most baseline measures used information from the T1 assessment, however, a small number of measures were added at T2 (see Tables) and for these, T2 was used as the baseline assessment. T3 data were always used for the final assessment measurements.

**Table 1:** Demographic and clinical characteristics of 149 ET participants.

		**Baseline assessment**	**Final assessment**
Demographic	Age (years)	78.7 ± 9.2 [80], (55–96)	81.7 ± 9.3 [83]
	Education (years)	15.8 ± 2.6 [16]	15.8 ± 2.6 [16]
	Sex (female)	93 (62.4)	93 (62.4)
	Race (white)	146 (98.0)	146 (98.0)
	Handedness (right)	126 (84.6)	126 (84.6)
Clinical	Age of tremor onset (years)	40.8 ± 22.1 [45], (5–89)	40.8 ± 22.1 [45]
	Tremor duration (years)	37.7 ± 21.7 [33], (2–86)	40.7 ± 21.7 [36]
	Number of medications that could impair balance and gait	0.79 ± 1.00 [1]	0.71 ± 0.88 [0]
Tremor Examination	Total Tremor Score	20.4 ± 4.7 [21]	19.8 ± 5.3 [20]
	Cranial Tremor Score	1.40 ± 1.10 [1]	1.15 ± 1.04 [1]
	Intention Tremor (present)	88 (59.1)	99 (66.4)
Cognition	MoCA	24.8 ± 3.2 [25]	23.0 ± 5.2 [24]
	CDR:
	0 (no dementia)	106 (71.1)	91 (61.5)
	0.5 (questionable dementia)	35 (23.5)	39 (26.4)
	1 (mild dementia)	8 (5.4)	10 (6.8)
	2 (moderate dementia)	0 (0)	6 (1.4)
	3 (severe dementia)	0 (0)	2 (1.4)
	Cognitive domain scores:
	Executive Function	0.03 ± 0.63 [0.12]	−0.06 ± 0.99 [0.14]
	Attention	−0.23 ± 0.77 [−0.30]	−0.25 ± 0.95 [−0.15]
	Visuospatial	0.47 ± 0.66 [0.47]	0.40 ± 0.83 [0.49]
	Primary cognitive diagnosis:
	Normal	117 (79.6)	109 (74.7)
	MCI	22 (15.0)	20 (13.7)
	Dementia	8 (5.4)	17 (11.6)
Sleep	ESS	6.9 ± 4.1 [6]	6.9 ± 4.1 [6]
Daily activity ability	IADL^a^	7.1 ± 1.8 [8]	6.7 ± 2.3 [8]
Depression	GDS-30	6.2 ± 5.0 [5]	7.3 ± 5.3 [7]
Anxiety	GAD-7^a^	2.8 ± 3.5 [2]	3.0 ± 3.8 [2]
Alcohol Usage	Average number of drinks per week	4.2 ± 8.7 [1]	2.8 ± 5.0 [0]
	Number of drinkers	73 (50.3)	63 (42.9)

CDR, Clinical Dementia Rating; ESS, Epworth Sleepiness Scale; GAD-7, General Anxiety Disorder Seven-Item; GDS-30, Geriatric Depression Scale Thirty-Item; IADL, Instrumental Activities of Daily Living; MCI, Mild Cognitive Impairment; MoCA, Montreal Cognitive Assessment.

*All values represent mean ± standard deviation [median], and (range); or number (percentage). T2 is baseline assessment^a^. When data were missing, total n was <149*.

**Table 2:** Gait and balance characteristics of ET participants.

		**Baseline assessment**	**Final assessment**	**Change from baseline to final assessment**	***P*-value for comparison between baseline and final assessment**	**Annual rate of change**
Performance-based tests	Tandem gait missteps	5.0 ± 4.1 [4]	6.2 ± 4.0 [7]	1.2 ± 3.7 [0]	***p*** **=** **0.000**^**a**^	0.4 ± 1.2 [0]
	Tandem stance time (seconds)^c^	6.1 ± 4.2 [8]	4.4 ± 4.2 [2]	−1.7 ± 4.0 [0]	***p*** **=** **0.000**^**a**^	−1.1 ± 2.6 [0]
Self-reported assessments	Near falls per year	18.9 ± 70.9 [0]	33.5 ± 79.1 [2.5]	14.6 ± 96.1 [0]	***p*** **=** **0.005**^**a**^	4.9 ± 32.0 [0]
	Falls per year	1.2 ± 2.7 [0]	1.7 ± 3.6 (1)	0.5 ± 3.6 [0]	*p* = 0.2^a^	0.2 ± 1.2 [0]
	Total ABC-6 score	336.9 ± 165.7 [360]	309.3 ± 166.5 [312.5]	−27.6 ± 109.2 [−15]	***p*** **=** **0.02**^**a**^	−9.2 ± 36.4 [−5]
	Used any type of walking aid^c^	38 (25.5)	62 (41.6)	24 (16.1)	***p*** **=** **0.000**^**b**^	16 (10.7)

ABC-6, Transformed Six-Item Activities of Balance Confidence Scale score.

*All values represent mean ± standard deviation [median], or number (percentage), and p-values. T2 is baseline assessment^c^. Wilcoxon Signed-Rank Test^a^. McNemar's Test^b^. Bolded values are significant (p < 0.05). For some tests, n < 149*.

All gait and balance measures at baseline and final assessments were tested for normality using the Kolmogorov-Smirnov test, which determined that each of the gait and balance parameters of interest were non-normally distributed; thus, non-parametric tests were used (Table 2) and all models were logistic rather than linear regressions (**Tables 3** and **4**).

In unadjusted logistic regression models, we examined associations between baseline clinical predictors of interest and five gait and balance outcomes *at the final (i.e., T3) assessment*; the outcome variables were coded as “more impaired” = 1 (e.g., more tandem missteps, less tandem stance time) vs. “less impaired” = 0 based on a median split (i.e., < median vs. > median) (Table 3A). In a second set of unadjusted logistic regression models, we examined the predictive utility of baseline clinical predictors of interest for *change* in five gait and balance parameters *over time*, which was defined as the T3 value subtracted by the baseline value; the outcome variables were coded as “greater decline in function” = 1 (e.g., increase in tandem gait missteps, decrease in tandem stance time) vs. “less decline in function” = 0 based on a median split (i.e., < median vs. > median) (Table 3B). In a third set of unadjusted logistic regression models, we examined associations between baseline clinical predictors of interest and five gait and balance outcomes at the final assessment (i.e., similar to those presented in Table 3A); however, in these analyses, the outcome variables were coded as “more impaired” = 1 (e.g., more tandem missteps, less tandem stance time) vs. “less impaired” = 0 *based on current benchmarks available in the ET literature rather than a median split* (i.e., based on available empiric data rather than a mathematical split, Table 3C). The cut-offs for impairment were as follows: ≥2 tandem gait missteps (8), ≥26 near falls per year (15), and ≤ 300 total ABC-6 score (15). All of the logistic regression analyses resulted in odds ratios (OR) with 95% confidence intervals (CI) and *p*-values. To adjust for multiple comparisons, significance for Table 3 was set at *p* < 0.01 in Tables 3A,B (Bonferroni correction because there were five outcome measures of interest) and *p* < 0.017 in Table 3C (because there were three outcome measures of interest). In Table 3D, we performed correlational analyses between baseline clinical predictors of interest and five gait and balance outcomes at the final (i.e., T3) assessment; these analyses used Spearman's correlation coefficients.

**Table 3A:** Unadjusted logistic regression models: Baseline characteristics as predictors of T3 gait and balance outcomes.

	**More tandem gait missteps (T3)**	**Less tandem stance time (T3)^**a**^**	**More near falls per year (T3)**	**Lower total ABC-6 score (T3)**	**Used any type of walking aid (T3)^**a**^**
**OR (95% CI)**					
Total tremor score	**1.12 (1.04–1.21)**	**1.12 (1.03–1.20)**	1.09 (1.01–1.18)	**1.11 (1.03–1.20)**	**1.14 (1.06–1.24)**
Tremor duration	1.01 (0.99–1.03)	1.02 (1.00–1.04)	1.00 (0.99–1.02)	1.01 (0.99–1.02)	1.01 (0.99–1.02)
Cranial tremor score	**1.72 (1.23–2.40)**	**1.79 (1.28–2.51)**	0.94 (0.69–1.28)	1.33 (0.97–1.83)	1.29 (0.96–1.74)
Intention tremor	2.39 (1.19–4.8)	1.62 (0.82–3.20)	1.21 (0.62–2.38)	1.37 (0.68–2.76)	1.32 (0.68–2.56)
MoCA	**0.75 (0.66–0.86)**	0.87 (0.77–0.97)	0.91 (0.82–1.02)	**0.75 (0.64–0.87)**	**0.86 (0.77–0.96)**
CDR	**13.60 (2.84–65.02)**	3.99 (1.04–15.35)	1.24 (0.35–4.39)	4.53 (1.10–18.69)	**11.60 (3.18–42.35)**
Executive function	**0.19 (0.09–0.39)**	0.51 (0.28–0.90)	0.67 (0.39–1.16)	**0.25 (0.12–0.51)**	**0.31 (0.17–0.57)**
Attention	**0.37 (0.23–0.62)**	0.72 (0.47–1.11)	0.82 (0.53–1.26)	**0.36 (0.21–0.62)**	**0.50 (0.31–0.79)**
Visuo–spatial	**0.44 (0.25–0.78)**	0.58 (0.34–0.98)	0.83 (0.81–1.38)	0.48 (0.27–0.85)	**0.42 (0.24–0.73)**
ESS	1.02 (0.94–1.11)	1.00 (0.92–1.09)	1.01 (0.93–1.10)	1.06 (0.97–1.16)	1.01 (0.93–1.10)
IADL^a^	**0.35 (0.17–0.68)**	**0.42 (0.24–0.76)**	1.04 (0.82–1.33)	0.59 (0.39–0.90)	**0.48 (0.33–0.72)**
GDS-30	1.09 (1.02–1.18)	**1.13 (1.04–1.22)**	1.06 (0.99–1.14)	**1.12 (1.03–1.21)**	1.04 (0.97–1.11)
GAD-7^a^	0.92 (0.81–1.04)	0.97 (0.87–1.09)	1.01 (0.91–1.13)	1.07 (0.95–1.20)	0.86 (0.74–1.00)
Average # of drinks per week	0.99 (0.95–1.03)	1.01 (0.97–1.05)	1.01 (0.97–1.05)	1.00 (0.95–1.05)	0.98 (0.94–1.02)
Number of drinkers	0.49 (0.25–0.99)	0.50 (0.25 – 1.00)	0.91 (0.46–1.79)	0.50 (0.25–1.01)	0.49 (0.25–0.96)

ABC-6, Transformed Activities of Balance Confidence Scale score; CDR, Clinical Dementia Rating; CI, Confidence Interval; ESS, Epworth Sleepiness Scale; GAD-7, General Anxiety Disorder Seven-Item; GDS-30, Geriatric Depression Scale Thirty-Item; IADL, Instrumental Activities of Daily Living; MoCA, Montreal Cognitive Assessment; OR, Odds Ratio.

All values represent odds ratio and 95% confidence interval.

a *T2 is baseline assessment. Bolded values are significant in the hypothesized direction (p < 0.01)*.

**Table 3B:** Unadjusted logistic regression models: Baseline characteristics as predictors of change in gait and balance outcomes.

	**Greater increase in tandem gait missteps**	**Greater decrease in tandem stance time^**a**^**	**Greater increase in near falls per year**	**Greater decrease in total ABC-6 score**	**Greater increase in use of any type of walking aid^**a**^**
**OR (95% CI)**					
Total tremor score	1.01 (0.94–1.09)	0.95 (0.87–1.03)	1.11 (1.02–1.20)	0.96 (0.89–1.03)	*0.89 (0.82–1.22)*
Tremor duration	0.99 (0.97–1.00)	1.00 (0.98–1.02)	1.00 (0.98–1.01)	1.01 (0.99–1.02)	1.00 (0.98–1.01)
Cranial tremor score	0.94 (0.68–1.30)	1.08 (0.76–1.52)	0.89 (0.65–1.22)	0.95 (0.70–1.31)	0.81 (0.60–1.08)
Intention tremor	2.28 (1.10–4.71)	1.20 (0.57–2.55)	1.17 (0.59–2.34)	0.48 (0.24–0.98)	0.81 (0.42–1.57)
MoCA	1.04 (0.93–1.16)	1.02 (0.91–1.17)	0.92 (0.82–1.03)	1.01 (0.90–1.13)	1.14 (1.02–1.27)
CDR	0.33 (0.08–1.31)	0.84 (0.17–4.25)	1.15 (0.32–4.11)	1.53 (0.41–5.68)	*0.13 (0.04–0.46)*
Executive function	1.23 (0.70–2.18)	0.96 (0.52–1.76)	0.64 (0.36–1.11)	0.89 (0.51–1.55)	*2.78 (1.55–5.00)*
Attention	1.01 (0.65–1.56)	1.01 (0.62–1.62)	0.91 (0.58–1.42)	1.09 (0.69–1.71)	*1.97 (1.25–3.11)*
Visuo-spatial	1.14 (0.67–1.93)	0.87 (0.49–1.54)	0.77 (0.46–1.28)	0.83 (0.48–1.42)	1.92 (1.14–3.25)
ESS	1.04 (0.95–1.13)	1.00 (0.91–1.10)	1.00 (0.91–1.09)	0.91 (0.83–1.00)	0.97 (0.90–1.06)
IADL^a^	1.12 (0.87–1.44)	1.28 (0.88–1.88)	1.08 (0.84–1.40)	0.94 (0.72–1.23)	*1.87 (1.29–2.69)*
GDS-30	0.98 (0.91–1.05)	1.10 (1.01–1.20)	1.03 (0.96–1.11)	1.00 (0.94–1.07)	0.96 (0.90–1.03)
GAD-7^a^	0.87 (0.75–1.00)	0.97 (0.86–1.09)	1.05 (0.94–1.18)	1.04 (0.93–1.17)	1.09 (0.96–1.23)
Average # of drinks per week	1.00 (0.97–1.04)	1.04 (0.98–1.09)	1.01 (0.98–1.05)	1.03 (0.98–1.09)	1.02 (0.98–1.06)
Number of drinkers	1.39 (0.69–2.80)	1.08 (0.51–2.28)	1.23 (0.61–2.44)	1.99 (0.99–4.03)	1.52 (0.79–2.92)

ABC-6, Transformed Activities of Balance Confidence Scale score; CDR, Clinical Dementia Rating; CI, Confidence Interval; ESS, Epworth Sleepiness Scale; GAD-7, General Anxiety Disorder Seven-Item; GDS-30, Geriatric Depression Scale Thirty-Item; IADL, Instrumental Activities of Daily Living; MoCA, Montreal Cognitive Assessment; OR, Odds Ratio.

All values represent odds ratio and 95% confidence interval.

a *T2 is baseline assessment. Underlined values are significant in the opposite of hypothesized direction (p < 0.01)*.

**Table 3C:** Unadjusted logistic regression models: Baseline characteristics as predictors of T3 gait and balance outcomes (based on available literature).

	**More tandem gait missteps (T3)**	**More near falls per year (T3)**	**Lower total ABC-6 score (T3)**
**OR (95% CI)**			
Total tremor score	1.12 (1.02–1.23)	1.05 (0.96–1.15)	**1.11 (1.03–1.20)**
Tremor duration	0.99 (0.97–1.01)	1.01 (0.99–1.03)	1.01 (0.99–1.02)
Cranial tremor score	1.14 (0.77–1.69)	0.81 (0.55–1.20)	1.35 (0.98–1.86)
Intention tremor	**9.71 (3.34–27.80)**	0.65 (0.30–1.47)	1.48 (0.74–2.98)
MoCA	**0.71 (0.58–0.86)**	**0.79 (0.69–0.91)**	**0.73 (0.63–0.85)**
CDR	5.27 (0.67–41.79)	**6.62 (1.64–26.77)**	4.83 (1.17–20.03)
Executive function	**0.26 (0.11–0.64)**	**0.36 (0.19–0.70)**	**0.24 (0.12–0.49)**
Attention	0.70 (0.41–1.20)	0.57 (0.33–1.00)	**0.38 (0.22–0.64)**
Visuo-spatial	0.42 (0.20–0.84)	0.64 (0.35–1.18)	0.49 (0.28–0.88)
ESS	0.98 (0.89–1.09)	1.03 (0.94–1.14)	1.06 (0.97–1.16)
IADL^a^	0.47 (0.21–1.09)	0.81 (0.63–1.05)	**0.58 (0.38–0.89)**
GDS-30	1.08 (0.98–1.20)	**1.11 (1.03–1.20)**	**1.12 (1.03–1.21)**
GAD-7^a^	0.91 (0.80–1.02)	1.12 (0.98–1.25)	1.07 (0.95–1.20)
Average # of drinks per week	1.02 (0.96–1.08)	0.99 (0.94–1.04)	1.01 (0.96–1.06)
Number of drinkers	0.53 (0.22–1.25)	0.91 (0.40–2.09)	0.53 (0.26–1.08)

ABC-6, Transformed Activities of Balance Confidence Scale score; CDR, Clinical Dementia Rating; CI, Confidence Interval; ESS, Epworth Sleepiness Scale; GAD-7, General Anxiety Disorder Seven-Item; GDS-30, Geriatric Depression Scale Thirty-Item; IADL, Instrumental Activities of Daily Living; MoCA, Montreal Cognitive Assessment; OR, Odds Ratio.

All values represent odds ratio and 95% confidence interval.

a *T2 is baseline assessment. Bolded values are significant in the hypothesized direction (p < 0.017)*.

**Table 3D:** Correlational analysis for logistic regression models.

	**Tandem gait missteps (T3)**	**Tandem stance time (T3)^**a**^**	**Near falls per year (T3)**	**Total ABC-6 score (T3)**	**Any type of walking aid (T3)^**a**^**
**[r**_**s**_, ***p*****-value]**					
Total tremor score	**0.27**, ***p*** **=** **0.001**	−0.19, *p* = 0.03	0.21, *p* = 0.01	−0.22, *p* = 0.01	**0.28**, ***p*** **=** **0.001**
Tremor duration	0.04, *p* = 0.64	−0.15, *p* = 0.09	0.07, *p* = 0.41	−0.11, *p* = 0.22	0.06, *p* = 0.45
Cranial tremor score	**0.22**, ***p*** **=** **0.009**	**−0.33**, ***p*** **=** **0.000**	−0.08, *p* = 0.38	−0.14, *p* = 0.11	0.13, *p* = 0.11
Intention tremor	**0.28**, ***p*** **=** **0.001**	−0.10, *p* = 0.26	0.02, *p* = 0.83	−0.14, *p* = 0.12	0.07, *p* = 0.42
MoCA	**−0.46**, ***p*** **=** **0.000**	**0.28**, ***p*** **=0.001**	−0.18, *p* = 0.04	**0.37**, ***p*** **=** **0.000**	**−0.23**, ***p*** **=** **0.005**
CDR	**0.29**, ***p*** **=** **0.001**	**−0.24**, ***p*** **=** **0.005**	0.06, *p* = 0.52	−0.24, *p* = 0.006	**0.30**, ***p*** **=** **0.000**
Executive function	**−0.46**, ***p*** **=** **0.000**	**0.29**, ***p*** **=** **0.001**	−0.22, *p* = 0.01	**0.42**, ***p*** **=** **0.000**	**−0.32**, ***p*** **=** **0.000**
Attention	**−0.31**, ***p*** **=** **0.000**	0.22, *p* = 0.01	−0.14, *p* = 0.11	**0.35**, ***p*** **=** **0.000**	**−0.27**, ***p*** **=** **0.001**
Visuo-spatial	**−0.25**, ***p*** **=** **0.003**	0.23, *p* = 0.008	−0.09, *p* = 0.31	**0.31**, ***p*** **=** **0.000**	**−0.24**, ***p*** **=** **0.004**
ESS	0.06, *p* = 0.54	−0.02, *p* = 0.81	0.02, *p* = 0.84	−0.02, *p* = 0.87	0.01, *p* = 0.90
IADL^a^	**−0.33**, ***p*** **=** **0.000**	**0.43**, ***p*** **=** **0.000**	−0.16, *p* = 0.09	**0.38**, ***p*** **=** **0.000**	**−0.41**, ***p*** **=** **0.000**
GDS-30	0.19, *p* = 0.03	−0.18, *p* = 0.04	**0.25**, ***p*** **=** **0.003**	**−0.27**, ***p*** **=** **0.002**	0.12, *p* = 0.16
GAD-7^a^	−0.15, *p* = 0.12	0.04, *p* = 0.66	0.12, *p* = 0.23	−0.07, *p* = 0.50	−0.16, *p* = 0.09
Average # of drinks per week	−0.13, *p* = 0.15	0.13, *p* = 0.15	0.01, *p* = 0.88	0.17, *p* = 0.06	−0.19, *p* = 0.02
Number of drinkers	−0.16, *p* = 0.07	0.17, *p* = 0.05	−0.001, *p* = 1.00	0.16, *p* = 0.08	−0.17, *p* = 0.04

ABC-6, Transformed Activities of Balance Confidence Scale score; CDR, Clinical Dementia Rating; ESS, Epworth Sleepiness Scale; GAD-7, General Anxiety Disorder Seven-Item; GDS-30, Geriatric Depression Scale Thirty-Item; IADL, Instrumental Activities of Daily Living; MoCA, Montreal Cognitive Assessment; r_s_, Spearman's correlation coefficient.

All values represent odds ratio and 95% confidence interval.

a *T2 is baseline assessment. Bolded values are significant in the hypothesized direction (p < 0.01)*.

We then considered the effects of potential confounding variables, specifically, age, medication that can impair gait and balance, education, and gender (Tables 4A–C). In adjusted model 1, we used a less restrictive approach to confounding—confounding variables were included in the model if they were associated with either the independent variable or the dependent variable. In adjusted model 2, we used a more restrictive model, and confounding variables were only included if they were associated with both the independent and dependent variables. Significance in Tables 4A–C were corrected for multiple comparisons, as they were in Tables 3A–C.

**Table 4A:** Adjusted logistic regression models: Baseline characteristics as predictors of T3 gait and balance outcomes.

		**More tandem gait missteps (T3)**	**Less tandem stance time (T3)^**j**^**	**More near falls per year (T3)**	**Lower total ABC-6 score (T3)**	**Used any type of walking aid (T3)^**j**^**
**OR (95% CI)**						
Total tremor score	Adjusted model 1	1.08 (0.99–1.18)^c^	1.08 (0.99–1.17)^a^	1.10 (1.02–1.19)^c^	**1.09 (1.00–1.18)**^**c**^	1.11 (1.02–1.21)^a^
	Adjusted model 2	1.09 (1.00–1.18)^a^	1.08 (0.99–1.17)^a^		**1.08 (1.00–1.18)**^**a**^	1.11 (1.02–1.21)^a^
Tremor duration	Adjusted model 1	1.00 (0.98–1.02)^c^	1.01 (0.99–1.03)^a^	1.00 (0.98–1.02)^b^	1.00 (0.98–1.02)^f^	1.00 (0.98–1.02)^a^
	Adjusted model 2					
Cranial tremor score	Adjusted model 1	1.64 (1.10–2.43)^c^	1.55 (1.07–2.24)^a^	0.98 (0.70–1.36)^e^	1.19 (0.82–1.72)^e^	0.99 (0.70–1.39)^d^
	Adjusted model 2	1.44 (1.00–2.07)^a^	1.55 (1.07–2.24)^a^		1.18 (0.84–1.65)^a^	1.03 (0.73–1.44)^a^
Intention tremor	Adjusted model 1	0.48 (0.22–1.08)^c^	0.75 (0.35–1.58)^a^	0.80(0.40–1.59)^b^	0.94(0.43–2.04)^f^	1.01 (0.48–2.11)^a^
	Adjusted model 2					
MoCA	Adjusted model 1	**0.78 (0.69–0.93)**^**c**^	0.93 (0.82–1.06)^a^	0.91 (0.81–1.02)^c^	**0.79 (0.68–0.92)**^**f**^	0.92 (0.82–1.04)^a^
	Adjusted model 2	**0.80 (0.69–0.93)**^**a**^	0.93 (0.82–1.06)^a^		**0.78 (0.67–0.91)**^**a**^	0.92 (0.82–1.04)^a^
CDR	Adjusted model 1	6.43 (1.20–34.49)^c^	1.75 (0.40–7.59)^c^	1.10 (0.30–4.11)^c^	2.21 (0.47–10.36)^f^	5.24 (1.30–21.17)^c^
	Adjusted model 2	6.43 (1.20–34.49)^c^	1.86 (0.43–8.14)^a^	1.09 (0.30–3.91)^b^	2.30 (0.52–10.30)^c^	**6.38 (1.59–25.61)**^**a**^
Executive function	Adjusted model 1	**0.26 (0.12–0.57)**^**f**^	0.71 (0.37–1.37)^f^	0.68 (0.37–1.24)^f^	**0.36 (0.17–0.78)**^**f**^	**0.40 (0.21–0.78)**^**f**^
	Adjusted model 2	**0.26 (0.12–0.56)**^**c**^	0.69 (0.37–1.28)^a^	0.72 (0.41–1.25)^b^	**0.36 (0.17–0.78)**^**f**^	**0.40 (0.21–0.75)**^**a**^
Attention	Adjusted model 1	**0.31 (0.17–0.55)**^**c**^	0.67 (0.41–1.08)^c^	0.88 (0.57–1.38)^b^	**0.35 (0.20–0.64)**^**f**^	**0.45 (0.27–0.76)**^**c**^
	Adjusted model 2	**0.41 (0.25–0.68)**^**b**^		0.88 (0.57–1.38)^b^	**0.40 (0.24–0.69)**^**b**^	
Visuo-spatial	Adjusted model 1	0.46 (0.24–0.87)^c^	0.60 (0.34–1.08)^a^	0.88 (0.53–1.47)^b^	0.59 (0.31–1.10)^f^	**0.40 (0.22–0.75)**^**a**^
	Adjusted model 2					
ESS	Adjusted model 1	1.05 (0.96–1.16)^c^	1.03 (0.94–1.13)^a^	1.00 (0.92–1.09)^b^	1.08 (0.98–1.19)^c^	1.04 (0.95–1.14)^a^
	Adjusted model 2					
IADL^j^	Adjusted model 1	0.42 (0.21–0.84)^c^	0.50 (0.27–0.93)^a^	1.04 (0.81–1.35)^c^	0.72 (0.48–1.07)^f^	**0.54 (0.36–0.81)**^**a**^
	Adjusted model 2	**0.38 (0.19–0.77)**^**a**^	0.50 (0.27–0.93)^a^		0.66 (0.43–1.01)^a^	**0.54 (0.36–0.81)**^**a**^
GDS-30	Adjusted model 1	1.11 (1.01–1.21)^c^	**1.14 (1.05–1.25)**^**a**^	1.05 (0.98–1.13)^b^	1.12 (1.03–1.22)^c^	1.04 (0.97–1.12)^a^
	Adjusted model 2					
GAD-7^j^	Adjusted model 1	0.94 (0.81–1.08)^c^	1.01 (0.89–1.15)^a^	1.02 (0.91–1.14)^b^	1.10 (0.98–1.25)^c^	0.88 (0.75–1.03)^a^
	Adjusted model 2					
Average # of drinks per week	Adjusted model 1	1.01 (0.97–1.06)^e^	1.03 (0.98–1.07)^g^	1.01(0.97–1.06)^h^	1.02 (0.96–1.08)^i^	0.99 (0.94–1.03)^g^
	Adjusted model 2					
Number of drinkers	Adjusted model 1	1.57 (0.70–3.50)^c^	0.56 (0.26–1.20)^a^	0.89(0.45–1.76)^b^	0.51(0.24–1.10)^f^	0.59 (0.28–1.23)^a^
	Adjusted model 2					

ABC-6, Transformed Activities of Balance Confidence Scale score; CDR, Clinical Dementia Rating; CI, Confidence Interval; ESS, Epworth Sleepiness Scale; GAD-7, General Anxiety Disorder Seven-Item; GDS-30, Geriatric Depression Scale Thirty-Item; IADL, Instrumental Activities of Daily Living; MoCA, Montreal Cognitive Assessment; OR, Odds Ratio.

Adjusted model 1: Less restrictive model for confounding [an association (p < 0.05) between the confounding variable and either the independent or dependent variable] Adjusted model 2: More restrictive model for confounding an association (p < 0.05) between the confounding variable and both the independent and dependent variable. If cell is empty, this signifies that no variables met requirements for confounders and that results are the same as those in the unadjusted models.

All values represent odds ratio and 95% confidence interval. All measures of time were recorded in seconds.

a Adjusted for age.

b Adjusted for medications that can decrease gait and balance.

c Adjusted for age and medications that can decrease gait and balance.

d Adjusted for age and gender.

e Adjusted for age, medications that can decrease gait and balance, and gender.

f Adjusted for age, medications that can decrease gait and balance, and years of education.

g Adjusted for age and gender.

h Adjusted for medications that can decrease gait and balance and gender.

i Adjusted for age, medications that can decrease gait and balance, education, and gender.

j *T2 is baseline assessment. Bolded values are significant in the hypothesized direction (p < 0.01)*.

**Table 4B:** Adjusted logistic regression models: Baseline characteristics as predictors of change in gait and balance outcomes.

		**Greater increase in tandem gait missteps**	**Greater decrease in tandem stance time^**j**^**	**Greater increase in near falls per year**	**Greater decrease in total ABC-6 score**	**Greater increase in use of any type of walking aid^**j**^**
**OR (95% CI)**						
Total tremor score	Adjusted model 1	1.02 (0.95–1.10)^c^	0.93 (0.85–1.02)^a^	1.11 (1.02–1.20)^c^	0.95 (0.88–1.02)^c^	0.91 (0.84–0.99)^a^
	Adjusted model 2	1.02 (0.94–1.10)^a^	0.93 (0.85–1.02)^a^		0.95 (0.88–1.03)^a^	0.91 (0.84–0.99)^a^
Tremor duration	Adjusted model 1	0.99 (0.97–1.01)^c^	1.00 (0.98–1.01)^a^	1.00 (0.98–1.01)^b^	1.01 (0.99–1.02)^f^	1.00 (0.99–1.02)^a^
	Adjusted model 2					
Cranial tremor score	Adjusted model 1	0.96 (0.68–1.35)^c^	1.00 (0.70–1.44)^a^	0.86 (0.61–1.20)^e^	0.95 (0.68–1.34)^e^	1.02 (0.73–1.43)^d^
	Adjusted model 2	0.97 (0.69–1.37)^a^	1.00 (0.70–1.44)^a^		0.94 (0.68–1.30)^a^	0.99 (0.71–1.38)^a^
Intention tremor	Adjusted model 1	0.42 (0.20–0.87)^c^	0.88 (0.41–1.88)^a^	0.85 (0.43–1.71)^b^	2.15 (1.04–4.48)^f^	0.96 (0.47–1.97)^a^
	Adjusted model 2					
MoCA	Adjusted model 1	1.03 (0.91–1.15)^c^	1.05 (0.92–1.20)^a^	0.93 (0.82–1.04)^c^	1.02 (0.90–1.15)^f^	1.06 (0.94–1.19)^a^
	Adjusted model 2	1.03 (0.91–1.15) ^a^	1.05 (0.92–1.20)^a^		1.01 (0.90–1.14)^a^	1.06 (0.94–1.19)^a^
CDR	Adjusted model 1	0.37 (0.09–1.54)^c^	0.65 (0.12–3.44)^c^	1.01 (0.28–3.77)^c^	1.50 (0.38–5.91)^f^	0.30 (0.08–1.16)^c^
	Adjusted model 2	0.37 (0.09–1.54)^c^	0.66 (0.13–3.50)^a^	1.16 (0.32–4.18)^b^	1.49 (0.38–5.84)^c^	0.24 (0.06–0.91)^a^
Executive function	Adjusted model 1	1.09 (0.58–2.05)^f^	1.33 (0.68–2.63)^f^	0.61 (0.33–1.13)^f^	0.88 (0.47–1.64)^f^	2.26 (1.17–4.33)^f^
	Adjusted model 2	1.13 (0.62–2.07)^c^	1.04 (0.56–1.94)^a^	0.62 (0.35–1.10)^b^	0.88 (0.47–1.64)^f^	2.19 (1.19–4.05)^a^
Attention	Adjusted model 1	0.98 (0.63–1.55)^c^	0.97 (0.59–1.60)^c^	0.90 (0.57–1.42)^b^	1.11 (0.69–1.77)^f^	*2.09 (1.26–3.44)^*c*^*
	Adjusted model 2	1.01 (0.65–1.57)^b^		0.90 (0.57–1.42)^b^	1.11 (0.70–1.78)^b^	
Visuo-spatial	Adjusted model 1	1.09 (0.63–1.86)^c^	0.90 (0.50–1.61)^a^	0.75 (0.45–1.27)^b^	0.82 (0.47–1.44)^f^	1.87 (1.06–3.31)^a^
	Adjusted model 2					
ESS	Adjusted model 1	1.04 (0.95–1.14)^c^	1.01 (0.92–1.11)^a^	0.99 (0.91–1.08)^b^	0.91 (0.82–1.00)^c^	0.91 (0.83–1.00)^a^
	Adjusted model 2					
IADL^j^	Adjusted model 1	1.08 (0.83–1.40)^c^	1.41 (0.94–2.11)^a^	1.11 (0.86–1.45)^c^	0.96 (0.72–1.28)^f^	**1.66 (1.15–2.39)**^**a**^
	Adjusted model 2	1.08(0.83–1.04)^a^	1.41 (0.94–2.11)^a^		0.96 (0.73–1.23)^a^	**1.66 (1.15–2.39)**^**a**^
GDS-30	Adjusted model 1	0.98 (0.92–1.05)^c^	1.10 (1.01–1.20)^a^	1.03 (0.96–1.11)^b^	1.00 (0.93–1.07)^c^	0.95 (0.89–1.03)^a^
	Adjusted model 2					
GAD-7^j^	Adjusted model 1	0.86 (0.75–0.99)^c^	0.98 (0.87–1.11)^a^	1.05 (0.94–1.18)^b^	1.05 (0.93–1.17)^c^	1.07 (0.94–1.22)^a^
	Adjusted model 2					
Average # of drinks per week	Adjusted model 1	0.99 (0.96–1.04)^e^	1.03 (0.98–1.09)^g^	1.01 (0.97–1.05)^h^	1.03 (0.98–1.10)^i^	1.01 (0.97–1.06)^g^
	Adjusted model 2					
Number of drinkers	Adjusted model 1	1.37 (0.67–2.80)^c^	1.16 (0.54–2.49)^a^	1.24 (0.62–2.47)^b^	2.03 (1.00–4.13)^f^	1.21 (0.59–2.50)^a^
	Adjusted model 2					

ABC-6, Transformed Activities of Balance Confidence Scale score; CDR, Clinical Dementia Rating; CI, Confidence Interval; ESS, Epworth Sleepiness Scale; GAD-7, General Anxiety Disorder Seven-Item; GDS-30, Geriatric Depression Scale Thirty-Item; IADL, Instrumental Activities of Daily Living; MoCA, Montreal Cognitive Assessment; OR, Odds Ratio.

Adjusted model 1: Less restrictive model for confounding [an association (p < 0.05) between the confounding variable and either the independent or dependent variable] Adjusted model 2: More restrictive model for confounding [an association (p < 0.05) between the confounding variable and both the independent and dependent variable. If cell is empty, this signifies that no variables met requirements for confounders and that results are the same as those in the unadjusted models.

All values represent odds ratio and 95% confidence interval. All measures of time were recorded in seconds.

a Adjusted for age.

b Adjusted for medications that can decrease gait and balance.

c Adjusted for age and medications that can decrease gait and balance.

d Adjusted for age and gender.

e Adjusted for age, medications that can decrease gait and balance, and gender.

f Adjusted for age, medications that can decrease gait and balance, and years of education.

g Adjusted for age and gender.

h Adjusted for medications that can decrease gait and balance and gender.

i Adjusted for age, medications that can decrease gait and balance, education, and gender.

j *T2 is baseline assessment. Underlined values are significant in the opposite of hypothesized direction (p < 0.01)*.

**Table 4C:** Adjusted logistic regression models: Baseline characteristics as predictors of T3 gait and balance outcomes (based on available literature).

		**More tandem gait missteps (T3)**	**More near falls per year (T3)**	**Lower total ABC-6 score (T3)**
**OR (95% CI)**				
Total tremor score	Adjusted model 1	1.07 (0.96–1.20)^c^	1.04 (0.95–1.14)^c^	1.09 (1.00–1.18)^c^
	Adjusted model 2	1.08 (0.97–1.20)^a^		1.08 (1.00–1.18)^a^
Tremor duration	Adjusted model 1	**0.97 (0.94–0.99)**^**c**^	1.01 (0.99–1.03)^b^	1.00 (0.98–1.02)^f^
	Adjusted model 2			
Cranial tremor score	Adjusted model 1	0.82 (0.50–1.34)^c^	0.84 (0.55–1.26)^e^	1.21 (0.83–1.75)^e^
	Adjusted model 2	0.79 (0.48–1.29)^a^		1.18 (0.84–1.66)^a^
Intention tremor	Adjusted model 1	**0.05 (0.01–0.22)**^**c**^	1.49 (0.65–3.40)^b^	0.86 (0.39–1.89)^f^
	Adjusted model 2			
MoCA	Adjusted model 1	**0.77 (0.62–0.95)**^**c**^	**0.79 (0.68–0.92)**^**c**^	**0.77 (0.66–0.91)**^**f**^
	Adjusted model 2	**0.76 (0.62–0.94)**^**a**^		**0.76 (0.65–0.89)**^**a**^
CDR	Adjusted model 1	1.31 (0.14–12.47)^c^	5.77 (1.34–24.80)^c^	2.29 (0.48–10.84)^f^
	Adjusted model 2	1.31 (0.14–12.47)^c^	**6.04 (1.48–24.64)**^**b**^	2.38 (0.53–10.75)^c^
Executive function	Adjusted model 1	0.41 (0.14–1.19)^f^	**0.40 (0.19–0.92)**^**f**^	**0.35 (0.16–0.76)**^**f**^
	Adjusted model 2	0.38 (0.13–1.08)^c^	**0.39 (0.20–0.75)**^**b**^	**0.35 (0.16–0.76)**^**f**^
Attention	Adjusted model 1	0.62 (0.32–1.21)^c^	0.62 (0.35–1.10)^b^	**0.37 (0.21–0.67)**^**f**^
	Adjusted model 2	0.78 (0.45–1.37)^b^	0.62 (0.35–1.10)^b^	**0.42 (0.25–0.72)**^**b**^
Visuo-spatial	Adjusted model 1	0.45 (0.20–1.02)^c^	0.69 (0.37–1.27)^b^	0.61 (0.32–1.15)^f^
	Adjusted model 2			
ESS	Adjusted model 1	1.02 (0.90–1.14)^c^	1.03 (0.93–1.13)^b^	1.08 (0.98–1.19)^c^
	Adjusted model 2			
IADL^j^	Adjusted model 1	0.65 (0.28–1.48)^c^	0.83 (0.64–1.08)^c^	0.71 (0.47–1.07)^f^
	Adjusted model 2	0.61 (0.26–1.42)^a^		0.65 (0.43–1.00)^a^
GDS-30	Adjusted model 1	1.08 (0.97–1.20)^c^	1.10 (1.02–1.19)^b^	**1.12 (1.03–1.21)**^**c**^
	Adjusted model 2			
GAD-7^j^	Adjusted model 1	0.94 (0.81–1.09), *p* = 0.40^c^	1.12 (0.99–1.27), *p* = 0.08^b^	1.11 (0.98–1.25), *p* = 0.11^c^
	Adjusted model 2			
Average # of drinks per week	Adjusted model 1	1.02 (0.96–1.09)^e^	0.98 (0.93–1.04)^h^	1.02 (0.97–5.17)^i^
	Adjusted model 2			
Number of drinkers	Adjusted model 1	0.52 (0.19–1.43)^c^	0.56 (0.24–1.28)^b^	0.56 (0.26–1.20)^f^
	Adjusted model 2			

ABC-6, Transformed Activities of Balance Confidence Scale score; CDR, Clinical Dementia Rating; CI, Confidence Interval; ESS, Epworth Sleepiness Scale; GAD-7, General Anxiety Disorder Seven-Item; GDS-30, Geriatric Depression Scale Thirty-Item; IADL, Instrumental Activities of Daily Living; MoCA, Montreal Cognitive Assessment; OR, Odds Ratio.

Adjusted model 1: Less restrictive model for confounding [an association (p < 0.05) between the confounding variable and either the independent or dependent variable] Adjusted model 2: More restrictive model for confounding an association (p < 0.05) between the confounding variable and both the independent and dependent variable. If cell is empty, this signifies that no variables met requirements for confounders and that results are the same as those in the unadjusted models.

All values represent odds ratio and 95% confidence interval. All measures of time were recorded in seconds.

a Adjusted for age.

b Adjusted for medications that can decrease gait and balance.

c Adjusted for age and medications that can decrease gait and balance.

d Adjusted for age and gender.

e Adjusted for age, medications that can decrease gait and balance, and gender.

f Adjusted for age, medications that can decrease gait and balance, and years of education.

g Adjusted for age and gender.

h Adjusted for medications that can decrease gait and balance and gender.

i Adjusted for age, medications that can decrease gait and balance, education, and gender.

j *T2 is baseline assessment. Bolded values are significant in the hypothesized direction (p < 0.017)*.

We also performed an additional analysis in which we stratified our sample into relatively younger (<70 years) vs. older ET cases, and presented data from the baseline and final assessments on numerous balance and gait measures (Table 5).

**Table 5:** Gait and balance differences stratified by age group.

			**Baseline assessment**	**Final assessment**	**Change from baseline to final assessment**	***P*–value for comparison between baseline and final assessment**	**Annual rate of change**
Younger group^*^ **(*****n*** **=** **25)**	Age		64.2 ± 4.6 [66]	67.1 ± 4.6 [69]			
	Performance–based tests	Tandem gait missteps	1.5 ± 2.5 [0.5]	3.0 ± 3.8 [1]	1.5 ± 3.3 [1]	***p*** **=** **0.04**^**a**^	0.5
		Tandem stance time (seconds)^c^	7.7 ± 3.6 [10]	6.2 ± 4.1 [7]	−1.5 ± 4.6 [0]	*p* = 0.3^a^	−1
	Self–reported assessments	Near falls per year	21.6 ± 45.8 [0]	13.8 ± 22.1 [3]	−7.8 ± 38.9 [0]	*p* = 0.8^a^	−2.6
		Falls per year	2.2 ± 5.3 [0]	1.2 ± 1.9 [1]	−1.0 ± 5.3 [0]	*p* = 0.9^a^	−0.3
		Total ABC−6 score	378.8 ± 160.8 [420]	372.8 ± 146.3 [360]	−6.0 ± 101.8 [−15]	*p* = 0.9^a^	−2
		Used any type of walking aid^c^	2 (8)	5 (20)	3 (12)	*p* = 0.3^b^	2
Older group^**^ **(*****n*** **=** **124)**	Age		81.6 ± 6.8 [81]	84.6 ± 6.9 [84]			
	Performance–based tests	Tandem gait missteps	5.5 ± 4.0 [5.5]	6.9 ± 3.7 [9]	1.4 ± 3.8 [0]	***p*** **=** **0.002**^**a**^	0.5
		Tandem stance time (seconds)^c^	5.8 ± 4.3 [6]	3.8 ± 4.1 [2]	−2.0 ± 3.8 [0]	***p*** **=** **0.000**^**a**^	−1.3
	Self–reported assessments	Near falls per year	18.0 ± 73.5 [0]	37.6 ± 85.3 [2.3]	19.6 ± 104.2 [0]	***p*** **=** **0.002**^**a**^	6.5
		Falls per year	0.99 ± 1.7 [0]	1.7 ± 3.9 [1]	0.7 ± 3.1 [0]	*p* = 0.1^a^	0.2
		Total ABC−6 score	317.7 ± 168.9 [310]	294.4 ± 168.1 [300]	−23.3 ± 110.6 [−15]	***p*** **=** **0.01**^**a**^	−7.8
		Used any type of walking aid^c^	36 (29)	57 (46)	21 (17)	***p*** **=** **0.000**^**b**^	14

ABC-6, Transformed Six-Item Activities of Balance Confidence Scale score.

All values represent mean ± standard deviation [median], or number (percentage), and p-values.

* Younger group consists of participants that were age 69 and under at baseline assessment.

** Older group consists of participants that were age 70 and above at baseline assessment.

a Wilcoxon Signed-Rank Test.

b McNemar's Test.

c *T2 is baseline assessment. Bolded values are significant (p < 0.05). For some tests, n < 25 or 124*.

## Results

There were 149 ET participants available for longitudinal analysis. At baseline, the mean age was 78.7 ± 9.2 [median = 80, range = 55–96] years (Table 1). Of these 149, 107 (71.8%) had one or more of the following features, qualifying them for a diagnosis of “ET-plus:” dystonia, rest tremor, intention tremor, MCI or dementia (Supplementary Table 1).

The mean ± standard deviation time elapsed between assessments T1 and T2 was 1.51 ± 0.19 [median = 1.5] years, and between T2 and T3, it was 1.49 ± 0.24 [median = 1.5] years. Between the baseline and final assessments, numerous balance and gait measures showed evidence of decline: more tandem missteps, fewer seconds in maintaining tandem stance, more near falls per year, lower total ABC-6 score (i.e., decline in balance confidence), and an increased proportion of participants using walking aids (Table 2). Annual rates of change in each gait and balance measure are also shown (Table 2).

In the first set of unadjusted logistic regression models, we examined associations between baseline clinical predictors of interest and five gait and balance outcomes at T3 (Table 3A). Baseline clinical predictors of interest were tremor (total tremor score and cranial tremor score), cognition (MoCA, CDR, executive function, attention, and visuo-spatial function), daytime sleepiness (ESS), activities of daily living (IADL), depression (GDS-30), anxiety (GAD-7), and average number of alcoholic drinks per week (Table 3A). Numerous baseline clinical predictors of interest were associated with greater number of tandem missteps on gait performance (e.g., higher total tremor score, higher cranial tremor score, lower MoCA, higher CDR, lower executive function, lower attention, lower visuospatial function), less tandem stance time (higher total tremor score, higher cranial tremor score, lower IADL, more depression), lower balance confidence (higher total tremor score, lower MoCA, lower executive function, lower attention, lower IADL, and more depression) and with use of a walking aid (e.g., higher total tremor score, lower MoCA, higher CDR, lower executive function, lower attention, lower visuospatial function) at T3 (Table 3A). We also performed correlational analyses between baseline clinical predictors of interest and five gait and balance outcomes at the final (i.e., T3) assessment (Table 3D), and the vast bulk of associations (21 of 25) in Table 3A remained significant. In adjusted models, many of these associations persisted between higher baseline tremor severity, more impaired baseline cognitive function, lower baseline IADL, more baseline depression and several of our outcomes of interest—greater tandem mis-steps, greater use of walking aid and lower balance confidence (Table 4A).

In the second set of unadjusted logistic regression models, we examined associations between baseline clinical predictors of interest and *change* in five gait and balance parameters. We only observed a paradoxical association between lower baseline tremor severity and better baseline cognitive function on the one hand and increase in use of any type of walking aid on the other hand (Table 3B). In adjusted models, there was an association between higher attention and higher baseline IADL and greater use of any type of walking aid (Table 4B).

In the third set of unadjusted logistic regression models, we examined baseline clinical predictors of gait and balance outcomes at T3, using cut-offs that had been established *in the literature* (i.e., tandem missteps, near falls per year, total ABC-6 score). In these analyses, lower MoCA and more executive dysfunction were associated with more tandem mis-steps; lower MoCA, higher CDR, and more executive dysfunction were associated with more near falls per year; higher total tremor score, lower MoCA, more executive dysfunction, more inattention and more depression were associated with lower balance confidence (Table 3C). In the adjusted models, many of these results were replicated (Table 4C).

We also performed an additional analysis in which we stratified our sample into relatively younger vs. older ET cases (Table 5), with respective mean ages of 64.2 and 81.6 years old at baseline. The younger group was small (*n* = 25); hence, it was statistically underpowered. Even with this, we saw a significant increase in tandem missteps over time. Declines in balance confidence and increased use of walking aids over time in this younger group did not reach statistical significance (Table 5). Interestingly, annual rate of change in tandem gait missteps and tandem stance time were strikingly similar across the two age groups (Table 5).

## Discussion

In this study, 149 elderly ET participants were evaluated prospectively and longitudinally to quantitatively assess change in gait and balance measures over time (i.e., natural history) and examine the relationship between baseline clinical predictors and changes in these gait and balance measures. To our knowledge, it is the only such study of its kind. Between the baseline and final assessments, numerous balance and gait measures showed evidence of decline and annual rates of changes were carefully quantified for each. We examined associations between baseline clinical predictors of interest and five gait and balance outcomes, with baseline lower MoCA and lower executive function standing out as the most consistently significant predictors of greater gait and balance impairment at follow up. Analyses that assessed changes in gait and balance parameters over time were less successful in terms of identifying meaningful baseline predictors of such change.

Previously published work has primarily been successful in establishing abnormalities in gait and balance in ET in cross sectional studies, sampling ET cases at one point in time. The comparison of 104 ET patients and 40 age-matched controls using gait analysis showed that ET patients demonstrated reduced gait speed, dynamic imbalance, and gait asymmetry during standard walk and clear impairment in tandem gait (58). Even at an advanced age, ET patients performed more poorly than controls. In another study that compared ET patients to age-matched PD patients, dystonia patients, and controls, patients with movement disorders had lower balance confidence, increased falls, and greater need for walking aids compared to controls (59). Further comparison between movement disorder groups revealed a stepwise severity trend for all measures with PD patients experiencing the most imbalance, then ET patients, followed by dystonia patients. The longitudinal data we now present are in agreement with our cross-sectional data, which showed that ET patients with lower cognitive scores had more gait impairments (60) and greater number of falls (14).

Our study is the first to use longitudinal data to quantitatively assess the natural evolution of gait and balance deficits over the course of ET. Our findings also indicate that there are baseline clinical features of ET, especially level of cognitive performance, that can predict the level of gait and balance function later in life. The implications of these results are that physicians may be able to identify which ET patients are at higher risk for dangerous falls. If this risk could be identified early, preventative interventions could be suggested, such as balance-focused physical therapy, in hopes of mitigating the consequence of ET-related gait and balance disorder later in life. These findings also add another dimension to the cognitive changes observed in ET; these changes can now be viewed as predictive of poorer gait and balance performance over time in ET.

The mechanistic basis for the gait and balance impairment in ET is not entirely clear. While aging and aging-related factors play a role, the gait impairment in ET has been shown to be in excess of that seen in age-matched controls, indicating that other factors are involved. In clinical studies as well as quantitative gait studies, ET results in significant impairments in gait speed, asymmetry, dynamic balance, and variability, which lead to functional consequences of increased falls risk. The impairments seen in ET are qualitatively similar to what has been reported in classic cerebellar ataxia, although present to a milder degree (8). This is not surprising given the links, both in neuroimaging and postmortem studies, between ET and changes in the cerebellum.

We should comment on the fact that in our models, we were able to identify more predictors of T3 gait and balance outcomes (Tables 3A,B, 4A,C) than of change in these gait and balance outcomes (Tables 3B, 4B). This could be related to the fact that the duration of follow up was <5 years and hence, change was modest in absolute terms. Additionally, because our outcome variables were not normally distributed, our models were logistic regressions rather than linear regressions; logistic regression forces all observations into one of two categories and hence may not be optimal for detecting more nuanced change over time.

We observed an association between baseline cognitive function in ET and poorer balance performance at follow-up. This raises the broader question, what is the prior literature linking baseline cognitive deficits with declines in gait and balance over time in the elderly? There is a robust literature that highlights the roles of executive function and attention in gait, and that deficits in these domains are associated with deterioration in gait in the elderly (22–24). There are similar data in PD (25). More specifically with respect to ET, in cross-sectional studies of ET, individuals with ET and lower cognitive test scores had impaired gait, lower balance confidence and a higher number of falls than their counterparts with ET and higher cognitive test scores (14). In another cross-sectional study of ET cases, the authors reported that more cognitive difficulty was associated with more tandem gait difficulty. The authors noted that walking requires the concurrent use of cognitive and motor neural systems and that it is possible that the cognitive and gait problems in ET reflect an underlying pervasive disorder affecting both cognitive and motor circuits (19).

We noted an interesting association between poorer baseline cognitive features and reduction in use of any type of walking aid over time (Table 3B). This may be due to the fact that use of such assist devices require the participant to understand and cognitively manage the device.

Several issues merit special comment and consideration. First, our study did not include age-matched control participants. However, the goal of these analyses was to collect and present natural history data rather than to test the hypothesis that decline in gait and balance measures in ET cases occurs at a rate that is greater than that seen in age-matched controls. The data that we presented on ET were therefore valid and they fill a gap in knowledge, even without a direct comparison group. The observation, from numerous cross-sectional studies of ET patients of various ages, that gait and balance are compromised in ET, however, is heavily suggestive that the rate of change is likely to be in excess of that seen in age-matched controls; however, our study design does not allow us to make this conclusion. It has been documented that problems with balance increase over time in the normal aging population. Two previous studies have found that 13% of patients at age 65 years, 35% at age 75 years, and 46% of patients at 85 years reported balance difficulties (61, 62). According to one study that investigated objective measures of gait and balance over a wide continuum of age ranges in healthy adults, there are significant age-related changes in measures such as tandem gait, steady state gait, and dual task gait, although age-stratified data were not provided in that study, making direct comparisons with our data impossible (63). Second, we recognize that our findings do not apply to all ET, as ours was an older cohort. This is an issue of generalizability rather than validity. Nonetheless, epidemiological studies indicate that the majority of prevalent ET cases are in older age groups, such as the one we studied, and hence, older individuals are the group of greatest interest. Nevertheless, it is valuable to assess gait and balance in a younger ET cohort. As such, we were able to perform an additional analysis in which we stratified our sample into relatively younger vs. older ET cases (Table 5), with respective mean ages of 64.2 and 81.6 years, and reported data on annual rates of change in these groups. The younger group was small; hence, it was statistically underpowered. Even so, we saw a significant increase in tandem missteps over time. The observable declines in balance confidence and increased use of walking aids over time in this younger group, however, did not reach statistical significance (Table 5). Future studies may wish to sample a larger cohort of younger ET cases so as to sample the other end of the age spectrum in ET.

Our findings are admittedly not without their limitations. Subjective measures of balance confidence are open to self-report bias, especially as cognition may decline between baseline and final assessments. As a related point, our assessment of gait and balance intentionally used clinically-grounded and functional measures which were possible in the field (i.e., in patient's homes), and which are more likely to be of clinical significance; we did not perform quantitative gait testing and such studies would have aided in the precision of our estimates of rates of change. Also, we did not perform imaging on our study subjects and this would have been of value in terms of studying the underlying structural correlates of some of our findings.

Yet, despite these limitations, our study also has several strengths, the most important being our novel longitudinal data. The comprehensive evaluation provided a wealth of clinical data across three time intervals, allowing us to assess a broad array of predictors as well as potential confounding factors.

We present a much-needed look into the course of disease for elderly patients with ET, focusing on the changes observed in gait and balance and the predictors of these changes. These findings present a useful tool for clinicians, patients, and their families to better understand and plan for changing disease-features over time.

## Data Availability Statement

The raw data supporting the conclusions of this article will be made available by the authors, without undue reservation.

## Ethics Statement

The studies involving human participants were reviewed and approved by the Yale University and Columbia University Internal Review Boards.

## Author Contributions

HD: acquisition of data, analysis and interpretation of data, drafting/editing manuscript, and final approval of work. MZ, KR, and TC: acquisition of data, drafting/editing manuscript, and final approval of work. AR, EH, and SC: drafting/editing manuscript and final approval of work. EL: conception and design of study, analysis and interpretation of data, drafting/editing manuscript, and final approval of work. All authors contributed to the article and approved the submitted version.

## Conflict of Interest

The authors declare that the research was conducted in the absence of any commercial or financial relationships that could be construed as a potential conflict of interest.
