# Supplementary material for: Prospective Longitudinal Study of Gait and Balance in a Cohort of Elderly Essential Tremor Patients
Source: Front Neurol. 2020 Nov 13;11:581703. doi: 10.3389/fneur.2020.581703 (PMC7691661; doi:10.3389/fneur.2020.581703)
Supplement: Supplementary file 1 [file Table_1.docx]

Supplementary Table 1: Demographic and clinical characteristics for diagnoses of ET and ET plus (ET with dystonia, intention tremor, rest tremor, MCI or dementia at baseline)

|  | | Entire ET cohort (n=149) | ET plus (n=107) |
| --- | --- | --- | --- |
| Demographic | Age (years) | 78.7 ± 9.2 [80] | 80.1 ± 9.5 [81] |
|  | Education (years) | 15.8 ± 2.6 [16] | 15.5 ± 2.8 [16] |
|  | Sex (female) | 93 (62.4) | 65 (60.7) |
|  | Race (white) | 146 (98.0) | 106 (99.1) |
|  | Handedness (right) | 126 (84.6) | 96 (89.7) |
| Clinical | Age of tremor onset (years) | 40.8 ± 22.1 [45] | 41.5 ± 22.2 [45] |
|  | Tremor duration (years) | 37.7 ± 21.7 [33] | 38.3 ± 22.2 [34] |
|  | Number of medications that could impair balance and gait | 0.79 ± 1.00 [1] | 0.84 ± 0.97 [1] |
| Tremor Examination | Total Tremor Score | 20.4 ± 4.7 [21] | 21.3 ± 4.6 [22] |
|  | Cranial Tremor Score | 1.4 ± 1.1 [1] | 1.5 ± 1.1 [1] |
|  | Intention Tremor (present) | 88 (59.1) | 88 (82.2) |
| Cognition | MoCA | 24.8 ± 3.2 [25] | 24.3 ± 3.4 [25] |
|  | CDR:   0 (no dementia)  0.5 (questionable dementia)  1 (mild dementia)  2 (moderate dementia)  3 (severe dementia) | 106 (71.1)  35 (23.5)  8 (5.4)  0 (0)  0 (0) | 69 (64.5)  30 (28.0)  8 (7.5)  0 (0)  0 (0) |
|  | Cognitive domain scores:  Executive Function  Attention  Visuospatial | 0.03 ± 0.63 [0.01]  -0.23 ± 0.77 [-0.30]  0.47 ± 0.66 [0.47] | -0.08 ± 0.66 [0.01]  -0.29 ± 0.77 [-0.35]  0.42 ± 0.62 [0.47] |
|  | Primary cognitive diagnosis:  Normal  MCI  Dementia | 117 (79.6)  22 (15.0)  8 (5.4) | 75 (71.4)  22 (21.0)  8 (7.6) |
| Sleep | ESS | 6.9 ± 4.1 [6] | 6.5 ± 3.9 [6] |
| Daily activity ability | IADL^1^ | 7.1 ± 1.8 [8] | 7.0 ± 1.6 [8] |
| Depression | GDS-30 | 6.2 ± 5.0 [5] | 6.3 ± 5.0 [5] |
| Anxiety | GAD-7^1^ | 2.8 ± 3.5 [2] | 2.4 ± 2.9 [1] |
| Alcohol Usage | Average number of drinks per week | 2.3 ± 3.0 [1] | 4.6 ± 9.5 [0.5] |
|  | Number of drinkers | 13 (52) | 60 (50) |
| CDR: Clinical Dementia Rating, ESS: Epworth Sleepiness Scale, GAD-7: General Anxiety Disorder Seven-Item, GDS-30: Geriatric Depression Scale Thirty-Item, IADL: Instrumental Activities of Daily Living, MCI: Mild Cognitive Impairment, MoCA: Montreal Cognitive Assessment. | | | |

All values represent mean ± standard deviation [median], or number (percentage). T2 is baseline assessment ^1^. When data were missing, total n was < 149.
